# The Endoplasmic Reticulum Chaperone GRP78/BiP Modulates Prion Propagation *in vitro* and *in vivo*

**DOI:** 10.1038/srep44723

**Published:** 2017-03-23

**Authors:** Kyung-Won Park, Gyoung Eun Kim, Rodrigo Morales, Fabio Moda, Ines Moreno-Gonzalez, Luis Concha-Marambio, Amy S. Lee, Claudio Hetz, Claudio Soto

**Affiliations:** 1 Mitchell Center for Alzheimer’s Disease and Related Brain Disorders, Department of Neurology, University of Texas Houston Medical School, 6431 Fannin St, Houston, TX 77030, USA; 2 Department of Obstetrics and Gynecology, Baylor College of Medicine, One Baylor Plaza, Houston, TX 77030, USA; 3 Universidad de los Andes, Facultad de Medicina. Av San Carlos de Apoquindo 2200, Las Condes, Santiago, Chile; 4 Department of Biochemistry and Molecular Biology, USC/Norris Comprehensive Cancer Center, Keck School of Medicine of the University of Southern California, USA; 5 Biomedical Neuroscience Institute, Faculty of Medicine, University of Chile, Santiago, Chile; 6 Center for Geroscience, Brain Health and Metabolism, Institute of Biomedical Sciences, University of Chile, Santiago, Chile; 7 Department of Immunology and Infectious diseases, Harvard School of Public Health, Boston MA, USA; 8 Buck Institute for Research on Aging, Novato, CA, 94945, USA

## Abstract

Prion diseases are fatal neurodegenerative disorders affecting several mammalian species, characterized by the accumulation of the misfolded form of the prion protein, which is followed by the induction of endoplasmic reticulum (ER) stress and the activation of the unfolded protein response (UPR). GRP78, also called BiP, is a master regulator of the UPR, reducing ER stress levels and apoptosis due to an enhancement of the cellular folding capacity. Here, we studied the role of GRP78 in prion diseases using several *in vivo* and *in vitro* approaches. Our results show that a reduction in the expression of this molecular chaperone accelerates prion pathogenesis *in vivo*. In addition, we observed that prion replication in cell culture was inversely related to the levels of expression of GRP78 and that both proteins interact in the cellular context. Finally, incubation of PrP^Sc^ with recombinant GRP78 led to the dose-dependent reduction of protease-resistant PrP^Sc^ *in vitro*. Our results uncover a novel role of GRP78 in reducing prion pathogenesis, suggesting that modulating its levels/activity may offer a novel opportunity for designing therapeutic approaches for these diseases. These findings may also have implications for other diseases involving the accumulation of misfolded proteins.

Prion diseases are fatal and transmissible neurodegenerative disorders that affect humans and animals^1^. They are characterized by spongiform brain degeneration, neuronal loss, and the accumulation of a pathogenic and infectious form of the prion protein (PrP^Sc^) generated at expenses of the normal/cellular prion protein (PrP^C^) (Ref. 2). Accumulation of misfolded prions is thought to exert their deleterious effects in cells by different signaling cascades. Compelling evidence indicates that endoplasmic reticulum (ER) stress is a hallmark event in prion diseases^3^,^4^.

The ER is an essential organelle that plays a key role in the maintenance of calcium homeostasis, lipid synthesis, as well as the synthesis and folding of secreted and membrane bound proteins^5^. Perturbation of physiological conditions in cells can induce ER stress, which triggers an adaptive reaction known as the unfolded protein response (UPR), aimed to restore cellular homeostasis or trigger apoptosis in irreversibly damaged cells^6^,^7^. The UPR controls the expression of a variety of genes involved in protein folding secretion and quality control^7^. ER stress has been reported in many different animal and cellular models of prion diseases^3^, in addition to patients affected with Creutzfeldt-Jakob disease^8^. ER stress has been proposed to have two main consequences on the progression of prion diseases: (i) it may contribute to neurological impairment due to repression of the synthesis of a cluster of synaptic proteins^9^, and (ii) it may operate as a signal to trigger neuronal loss^8^,^10^. Importantly, accumulating evidence indicates that the contribution of the UPR to neurodegeneration is complex and largely depends on the UPR signaling branch affected and the disease context^11^.

The ER chaperone GRP78 (78-kDa glucose regulated protein), also referred to as BiP (Binding immunoglobulin protein) or HSPA5 (heat shock protein family A, member 5), is considered an essential ER chaperone and a master regulator of ER homeostasis^12^. GRP78 facilitates folding and assembly of nascent polypeptides, prevents their misfolding and aggregation, targets misfolded proteins for proteasome degradation, and controls the signaling for the initiation of the various arms of the UPR^13^,^14^. GRP78 operates as a repressor of UPR stress sensors through direct binding to them^7^. GRP78 has also been shown to have a chaperone activity by directly binding and preventing the aggregation of misfolded proteins in the ER^15^,^16^. In addition, GRP78 has multiple functions in cell signaling beyond its role in protein folding and has been found in various subcellular locations besides of the ER (reviewed in ref. 17). Considering its multiple functions, GRP78 has been described to actively participate in a wide variety of physiological and pathological processes^18^,^19^.

With respect to prion induced pathology, we and others showed that GRP78 levels were significantly increased in cells treated with PrP^Sc^ (refs 8 and 20), as well as transiently in prion infected mice^21^. Importantly, brains from patients affected by sporadic and variant Creutzfeldt-Jakob disease also showed increased levels of this particular chaperone^8^. GRP78 has been reported to physically interact with mutant PrP and mediate its degradation by the proteasome^15^, providing evidence that GRP78 is chaperoning the folding of PrP. Until now, only *in vitro* evidence and correlative studies in mouse models suggest a possible role of GRP78 in prion diseases. However, its actual contribution to prion pathogenesis remains unexplored. In this study, we examined the impact of targeting GRP78 in prion-induced pathology in animal models, as well as in genetically modified cell cultures. Our data shows that the reduction of GRP78 *in vivo* accelerates prion replication, thus resulting in a decreased incubation time of the disease. Additionally, we show that GRP78 over-expression reduces PrP^Sc^ levels in CAD5 cells infected with scrapie prions, whereas knocking down GRP78 by treatment with siRNA significantly increases prion replication. Immunocytochemistry and co-immunoprecipitation studies suggest that GRP78 and PrP^C^ directly interact in cells. Moreover, *in vitro* experiments using recombinant GRP78 show that this chaperon is able to disassembly PrP^Sc^ in a dose-dependent manner. Our findings indicate that GRP78 plays a key protective role in preventing the propagation of infectious prions, suggesting that the ER proteostasis network is implicated in prion diseases.

## Results

### *In vivo* reduction of GRP78 expression accelerates prion disease

**Figure 1:**
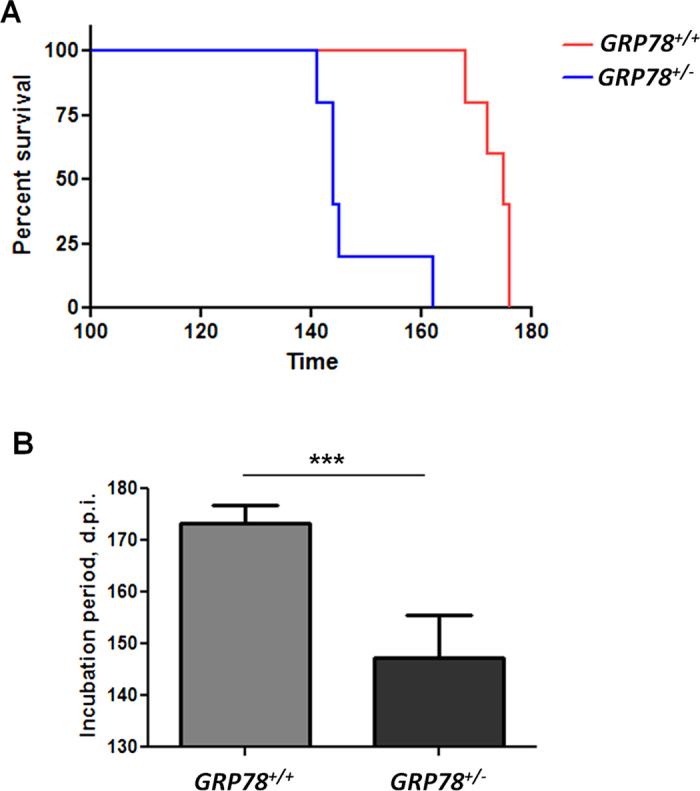
Decreased levels of GRP78 accelerate prion disease in mice. (**A**) Survival curve of *GRP78* heterozygous (*GRP78*^+/−^) and wild type (*GRP78*^+/+^) mice intra-cerebrally inoculated with RML prions. Differences in animal survival were analyzed by the Log-rank (Mantel Cox) test and found highly significant (P = 0.0018). (**B**) Average incubation periods of the different groups showed in panel A. Data is expressed as averages ± standard errors. Differences among the groups were analyzed by student’s t-test. ***P < 0.001.

To study the possible involvement of GRP78 in prion disease *in vivo*, we used a mice model heterozygous for *GRP78 (GRP78*^+/−^). Complete knock out mice of this important chaperone are not viable, whereas heterozygous mice are viable and develop normally^22^, although they have been shown to suffer from haplo-insufficient phenotypes under pathological conditions such as cancer^23^. *GRP78*^+/−^ and control (*GRP78*^+/+^) mice of the same strain were inoculated with the Rocky Mountain Laboratory (RML) strain of murine adapted scrapie prions. We directly injected these animals with RML prions into the brain and monitored disease progression and animal survival. Remarkably, *GRP78*^+/−^ mice were highly susceptible to prion infection, resulting on an acceleration of the disease when compared with *GRP78*^+/+^ littermate controls (Fig. 1A). The median survival time for *GRP78*^+/−^ mice was 144 days-post-inoculation (d.p.i), whereas *GRP78*^+/+^ mice survived for 175 d.p.i (Fig. 1B). Differences between both groups were statistically significant (*p* < 0.001). These data indicate that a reduction in GRP78 levels substantially accelerates the pathogenesis of prion diseases, suggesting an important protective role of this chaperone in this pathology.

### End-point prion disease characteristics are not altered by lower levels of GRP78 expression

**Figure 2:**
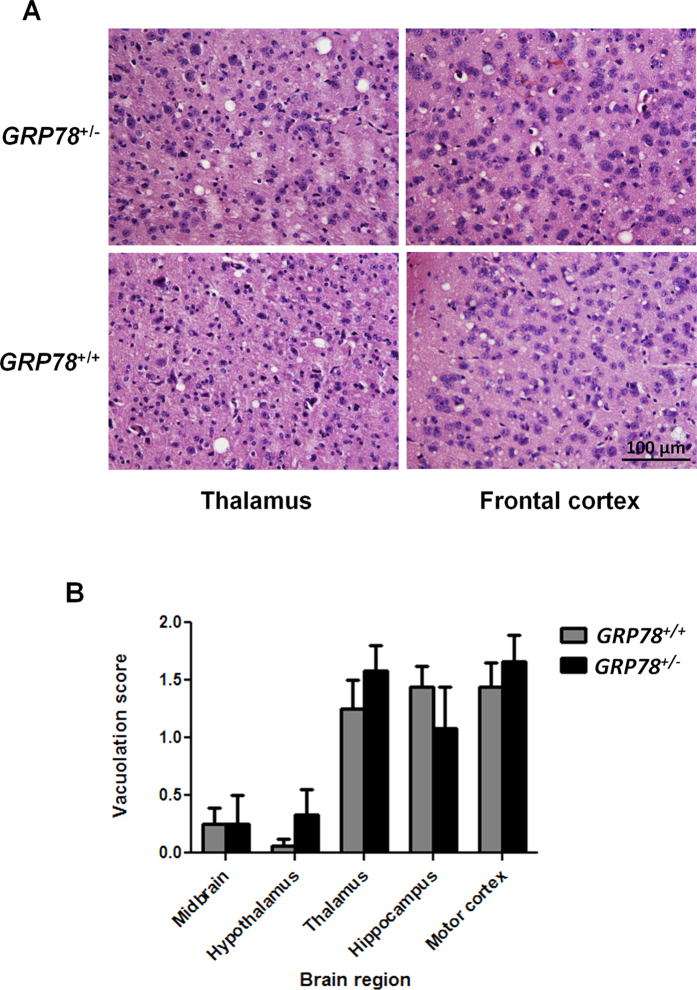
*GRP78* expression does not alter the vacuolation profile of terminally ill prion infected mice. (**A**) Thalamus and frontal cortex sections of brains from RML-symptomatic *GRP78* heterozygous (*Grp78*^+/−^) and wild type (*Grp78*^+/+^) mice were analyzed histologically for spongiform degeneration after hematoxylin-eosin staining. Bar in the lower right panel depict 100 μm and is representative of all pictures in this panel. (**B**) The vacuolation lesion profiles were determined on H&E stained sections from 5 different animals in each group. Degree of vacuolation was analyzed by scoring midbrain; hypothalamus; thalamus; hippocampus and motor cortex.

We then characterized different histopathological features of prion disease on our RML-infected mice. We analyzed the brain vacuolation profile in each group, as previously described^24^,^25^. Brain coronal sections stained with hematoxylin/eosin (H&E) showed the characteristic lesions expected for RML prions in thalamus and frontal cortex (Fig. 2A). The average lesion profile in various brain regions for both *GRP78*^+/+^ and *GRP78*^+/−^ mice was indistinguishable (Fig. 2B). We observed that the degree and proportion of vacuolation in both animal groups was the same at the end disease stage, regardless of the different incubation times observed. These results suggest that the faster appearance of clinical signs in *GRP78*^+/−^ mice was not due to a differential targeting of prions to specific brain structures. Additionally, we did not observe differences in PrP^Sc^ accumulation in the brain of *GRP78*^+/−^ and *GRP78*^+/+^ animals as assessed by immunostaining at the terminal stage of the disease (data not shown).

### Expression levels of ER chaperones and stress proteins in prion infected *GRP78* ^+/−^ and *GRP78* ^*+/+*^ mice

**Figure 3:**
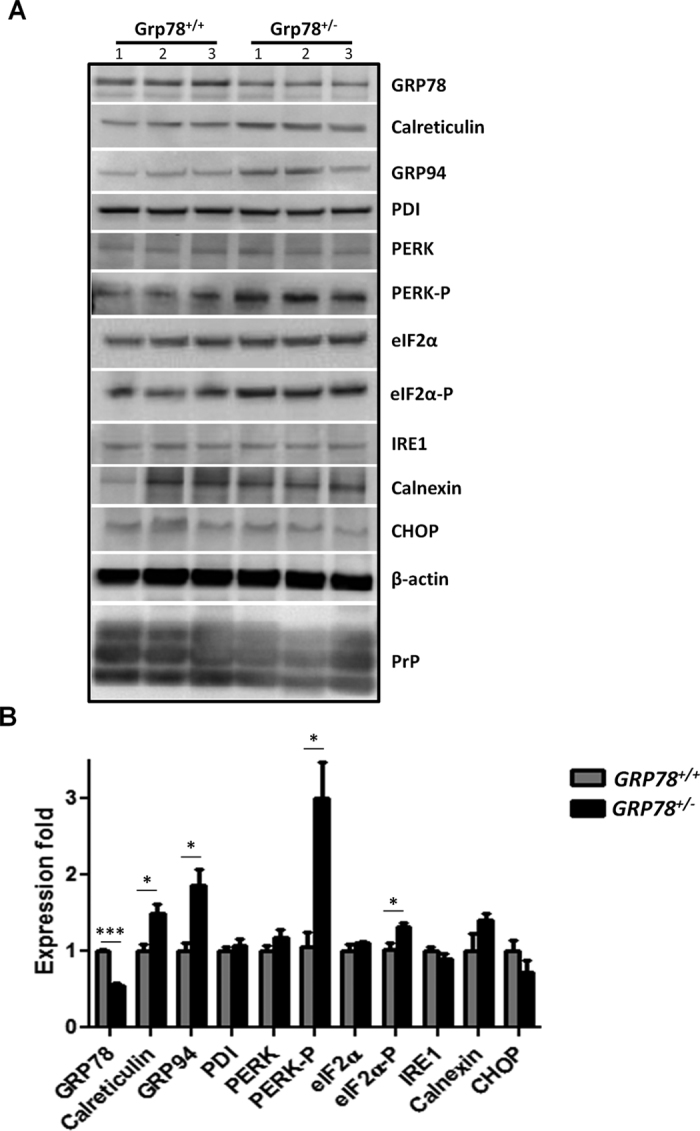
Expression levels of ER stress-responsive proteins in prion infected *GRP78*^+/+^ and *GRP78*^+/−^ mice. (**A**) Protein expression levels of UPR signaling proteins and ER chaperones from brains of *GRP78*^+/+^ and *GRP78*^+/−^ mice infected with prions were examined by Western blot. Proteins analyzed included GRP78, GRP94, PDI, IRE1, CHOP, PERK, eIF2α, calnexin, calreticulin as well as the phosphorylated forms of PERK and eIF2α. PrP^Sc^ content was assessed after treating brain extracts with PK as explained in Experimental Procedures. β-actin is shown as a loading control. Numbers at the top of the panel represent samples from three different animals in each group. For all samples, the same amount of total protein was loaded in each lane. For space constrains blots were cropped, but all samples were run using the same conditions and in the same gel. (**B**) Quantifications of Western blot signals from 3 replicates of the experiment shown in panel A are represented as averages ± standard errors. Statistical differences were analyzed by using student’s t-test. *P < 0.05, ***P < 0.001.

Next, we analyzed the expression of chaperones, ER-stress targets and proteins involved in the UPR in prion infected *GRP78*^+/−^ and *GRP78*^+/+^ mice to investigate whether the reduction of GRP78 exacerbates ER stress levels in prion-infected mice. As expected, we found that GRP78 protein levels in *GRP78*^+/−^ brains were reduced by about 50% when compared to those of control littermates (Fig. 3A). On the contrary, the expression levels of GRP94 and calreticulin were increased by ~1.5 and ~1.7 fold, respectively, in *GRP78*^+/−^ mice, suggesting compensatory changes to reduce protein folding stress (Fig. 3B). Other ER chaperones, such as calnexin and PDI did not show any significant differences between *GRP78*^+/−^ and *GRP78*^+/+^ infected mice (Fig. 3). Although the expression levels of other UPR targets, such as PERK, IRE1, eIF2α and CHOP, were not altered in *GRP78*^+/−^ mice brains when compared to the *GRP78*^+/+^ counterparts, the levels of PERK-P and eIF2α-P were increased by ~3 and ~1.4 fold, respectively, in *GRP78*^+/−^ mice (Fig. 3B). These results suggest that reduction of GRP78 during prion infection does not generate a global alteration in the ER proteostasis network, but might induce changes on specific UPR targets, particularly PERK-P and eIF2α-P, which may lead to significantly reduced survival of prion infected mice. This conclusion is consistent with recent reports indicating a key role of PERK-P and eIF2α-P in prion-induced neurodegeneration^9^,^26^.

We also monitored the levels of PrP^Sc^ at the terminal stage of the disease using proteinase K (PK) assays followed by Western blot analysis. The levels of PrP^Sc^ were similar in all mice regardless of the incubation periods (Fig. 3A), suggesting that although misfolded prion accumulation was accelerated, the end levels of the abnormal protein were not altered by reduced *GRP78* expression.

### GRP78 interacts with PrP^C^

**Figure 4:**
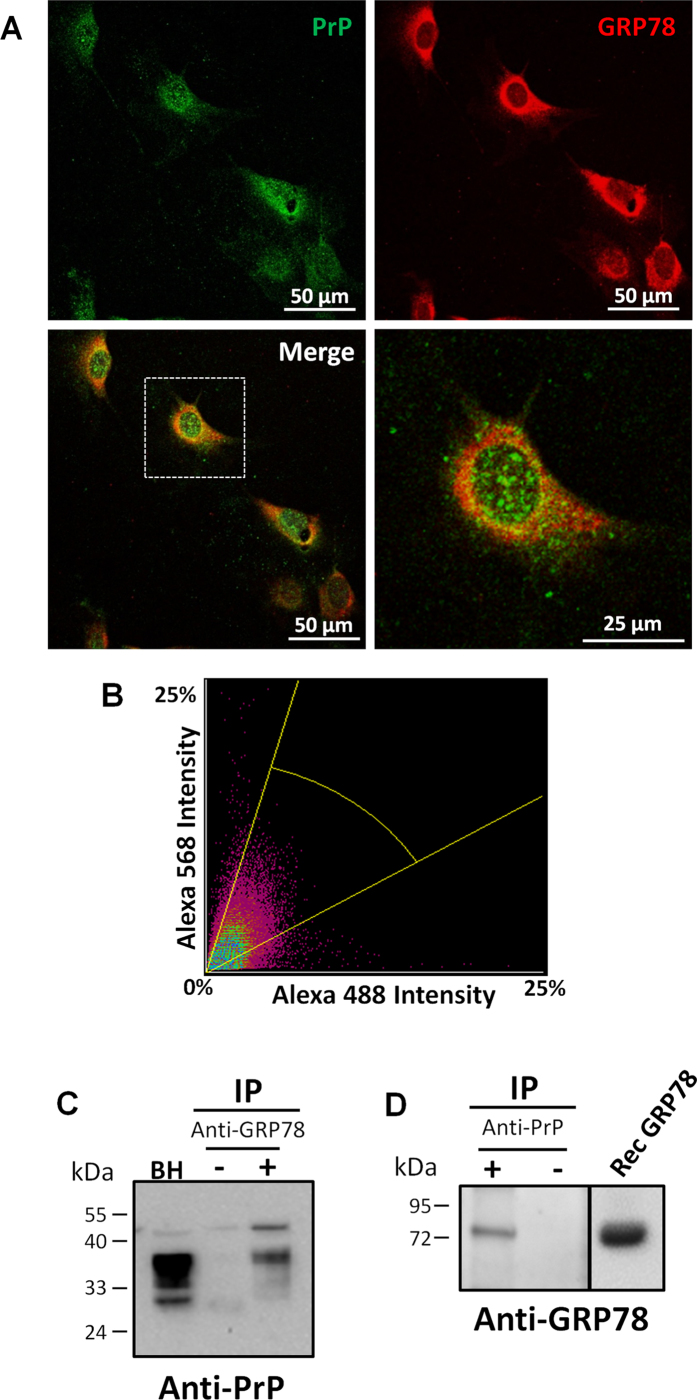
GRP78 interacts with PrP. (**A**) Primary cultures of mouse fibroblasts were doubly labeled with antibodies against PrP and GRP78 proteins. Top left panel represents cells that have been labeled with the 6H4 anti-PrP antibody and detected with Alexa488 secondary antibody (green). Top right panel represents cells that have been stained with anti-GRP78/BiP and detected with Alexa568 secondary antibody (red). Bottom left panel represents the merge between the two staining. Bottom right panel is a zoomed picture of one cell of the merged pictures (depicted in the dotted box in the bottom left panel). Samples were visualized by a confocal microscope. Scale bar: 50 μm or 25 μm. (**B**) Representative fluorogram indicating the signal intensity for both stainings and the colocalization of 6H4 (Alexa 488) and GRP78 (Alexa 568) obtained from confocal images. (**C**) Wild type mouse brain homogenates were immunoprecipitated with the anti-GRP78 antibody. Samples were analyzed by Western blot using an anti-PrP antibody (6D11). Lane 1 represents untreated brain homogenates used as a control, lane 2 corresponds to precipitation done with uncoated beads (without anti-GRP78 antibody), and lane 3 represents the immunoprecipitation with anti-GRP78 antibody. (**D**) Wild type mouse brain homogenates were immunoprecipated with the 6D11 anti-PrP antibody and samples analyzed by Western blot with anti-GRP78 antibody. First lane corresponds to the immoprecipitation with the 6D11 antibody, whereas the second line is the precipitation with the beads alone. Third lane depicts recombinant GRP78. Numbers on the left side of the gels correspond to the molecular weight standards. Separation line in the right blot indicate gel splicing to remove some irrelevant lines, even though all the samples were run in the same gel.

Since PrP^C^ is synthesized and modified in the ER (including disulfide bond formation, N-linked glycosylation, and GPI-anchor addition), we examined whether GRP78 may directly bind to this protein. We first performed immunocytochemistry experiments in primary cultures of wild type, non-infected, mouse fibroblasts. PrP was stained by using the 6H4 monoclonal antibody, followed by secondary antibody labeled with Alexa488 (in green). Staining was seen in the cytoplasm, the perinuclear compartment, and the cell surface (Fig. 4A, top left panel). GRP78 was stained by a specific antibody against this protein followed by the respective secondary antibody labeled with Alexa568 (in red) and showed a similar sub-cellular localization as PrP (Fig. 4A, top right panel). When the double labeling of both the anti-PrP and anti-GRP78 antibodies was examined simultaneously, there was a substantial blending of the immuno-reactivity merge, suggesting co-localization of both proteins (Fig. 4A, bottom panels). Co-localization analysis was performed to quantify the pixel co-distribution of 6H4 and anti-GRP78 antibodies using images obtained in a confocal microscope (Fig. 4B). The Pearson correlation coefficient (0.509 ± 0.037) demonstrated a good co-localization between GRP78 and PrP (1 = perfect correlation, 0 = no correlation, and −1 = perfect inverse correlation). In addition, Mander’s overlap coefficient (0.838 ± 0.044) also indicated that the 6H4 and GRP78 signals co-localize in the cell. The two-dimensional histogram for the distribution of pixel intensities for 6H4 and GRP78 reveals a positive spatial correlation (Fig. 4B).

To further study a possible interaction between PrP^C^ and GRP78, co-immunoprecipitation experiments were done with brain homogenates prepared from wild type mice. PrP^C^ was efficiently precipitated with the anti-GRP78 antibody (Fig. 4C, lane 3), whereas no signal was detected after incubation with anti-rabbit IgG Dynabeads alone (Fig. 4C, lane 2). Similarly, GRP78 was co-immunoprecipitated with anti-PrP antibodies, but not with beads alone (Fig. 4C). Altogether, these results indicate that PrP^C^ and GRP78 directly interact inside cells.

### GRP78 expression modifies PrP^Sc^ replication in prion infected cells

**Figure 5:**
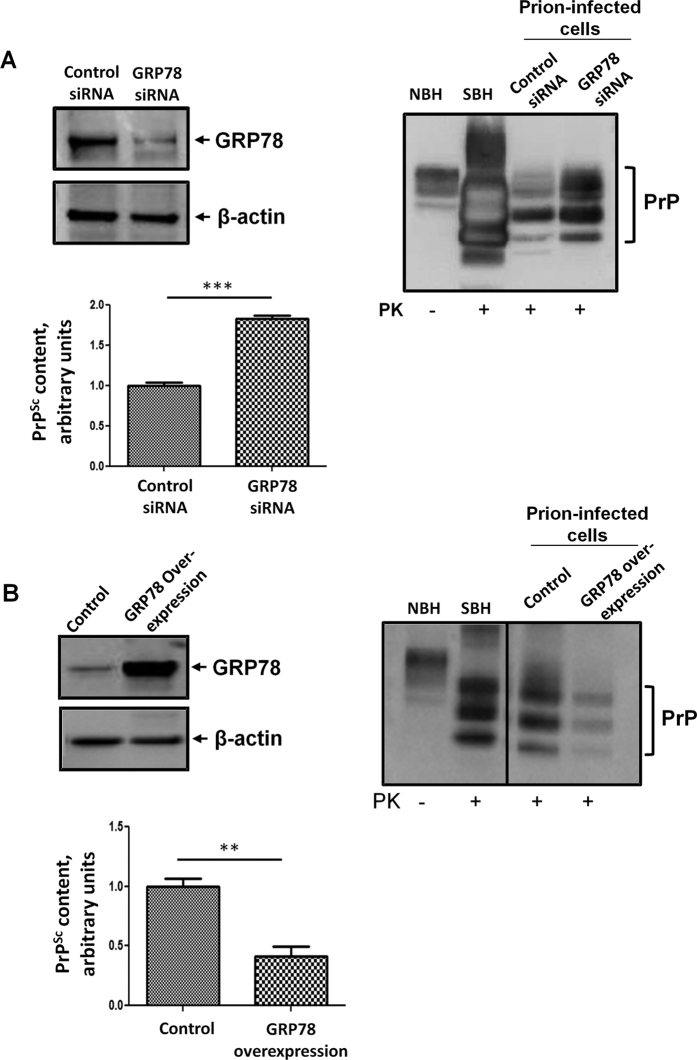
Expression levels of GRP78 modify prion replication in chronically infected CAD5 cells. (**A**) Prion-infected CAD5 cells transfected with *GRP78* siRNA or control siRNA were harvested and lysed. The expression of GRP78, actin (loading control), and PrP^Sc^ was analyzed by Western blotting. Left blot shows the staining with GRP78 antibody. Right blot corresponds to the staining with anti-PrP antibody. The graph shows the densitometric analysis of the levels of PrP^Sc^ in cells treated with control or GRP78 siRNA. (**B**) Prion-infected CAD5 cells transfected with *GRP78* overexpressing plasmid or control plasmid were harvested and lysed. The expression of GRP78, actin, and PrP^Sc^ was analyzed by Western blotting. Left blot depicts the staining for GRP78. Right blot corresponds to the staining for PrP. In this panel, the vertical line indicates gel splicing to remove some irrelevant lanes, but samples were run in the same gel and were developed with the same exposition. The graph shows the densitometric analysis of PrP^Sc^ levels in cells expressing endogenous amounts of *GRP78* (control) or over-expressing this protein. In both panels A and B, NBH: normal brain homogenate, not treated with PK, used as a marker of PrP^C^ migration. SBH: RML-infected brain homogenate treated with PK, used as a marker of protease-resistant PrP^Sc^ migration. For space constrains some blots were cropped, but all samples were run using the same conditions and in the same gel. Statistical differences were analyzed by using student’s t-test. **P < 0.01, ***P < 0.001.

To further study the functional role of GRP78 in prion replication, we performed studies with a CAD5 cell line chronically infected with mouse prions^27^. *GRP78* expression was experimentally altered in these cells in order to investigate whether this protein modulates the formation of PrP aggregates. We performed gain- and loss-of function studies by overexpressing the *GRP78* gene, or by silencing its expression using siRNAs. Modified cells were lysed and GRP78 and PrP^Sc^ levels monitored by western blot analysis. Remarkably, immunoblotting revealed that reduction of *GRP78* using siRNA significantly increased PrP^Sc^ accumulation in CAD5 cells (Fig. 5A), whereas overexpression of *GRP78* decreased the accumulation of the disease associated prion isoform (Fig. 5B). These results suggest that PrP^Sc^ replication is altered by changes in *GRP78* expression, confirming the *in vivo* results shown above. The effect of GRP78 was not at the level of PrP^C^ expression, since non-infected cells treated with siRNA to reduce GRP78 expression showed no differences in the levels of PrP^C^ (Supplementary Figure 1).

### GRP78 reduces protease-resistance of PrP^Sc^ in cell-free systems

**Figure 6:**
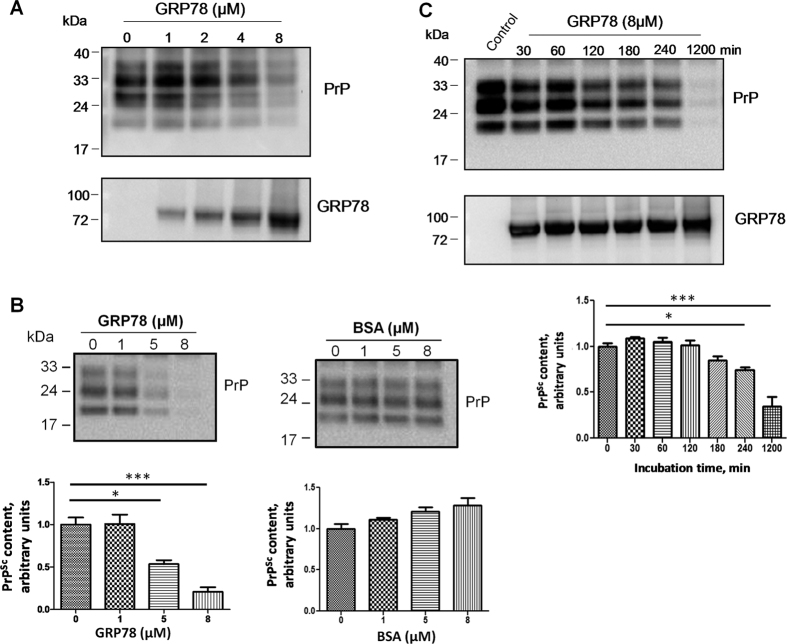
GRP78 reduces the amount of protease-resistant PrP^Sc^ *in vitro*. (**A**) RML infected brain homogenates were incubated with different concentrations of purified recombinant GRP78 for 20 h (1200 minutes), and the amount of PrP^Sc^ remaining resistant to PK digestion was assessed by Western blotting (top panel). Numbers at the top represent GRP78 concentrations, expressed as μM. (**B**) Highly purified PrP^Sc^ preparations were incubated with different concentrations of GRP78 for 20 h, and reactions were analyzed by Western blotting. As a control, BSA (bovine serum albumin) was used at the same concentrations. Numbers at top of each gel represent μM protein concentration supplemented in each case. Graphs below each blot represent the densitometric analysis of 3 replicates for each respective experiment. Values correspond to the average ± standard error and differences were analyzed by one-way ANOVA followed by Tukey’s multiple comparison test. (**C**) Purified PrP^Sc^ preparations were incubated with 8 μM of GRP78 for different time points ranging from 30 to 1200 minutes. “Control” represents a purified PrP^Sc^ aliquot without any treatment. The graph below represents the densitometric analysis of 3 replicates showed as the average ± standard error. Differences were analyzed by one-way ANOVA followed by Tukey’s multiple comparison test. All samples used to evaluate PrP^Sc^ signal were first treated with PK. Immunoblot was used to assess remaining protease-resistant PrP^Sc^ in each case. *P < 0.05; ***P < 0.001.

Besides its role as a master regulator of the UPR, GRP78 is a chaperone protein that binds hydrophobic residues in unfolded or partially folded proteins^28^. GRP78 was shown before to physically associate with mutant PrP^15^. To examine whether GRP78 may directly affect PrP^Sc^, brain homogenates of RML-infected mice containing large quantities of pre-formed PrP^Sc^ aggregates were incubated with purified recombinant GRP78. After incubation, we monitored the levels of PrP^Sc^ by employing an assay that takes advantage of the classical property of PrP^Sc^ of being highly resistant to proteolytic degradation. We observed a dose-dependent reduction in the levels of protease-resistant PrP^Sc^ in extracts incubated with different concentrations of recombinant GRP78 (Fig. 6A). Although these results suggest a direct effect of GRP78 on PrP^Sc^, an indirect activity of GRP78 over other putative molecules supporting the PrP^Sc^ structure cannot be discarded. To study the possible effects of GRP78 over PrP^Sc^ in a more direct manner, highly purified PrP^Sc^ was incubated with different concentrations of recombinant GRP78. We found that 8 μM of recombinant GRP78 almost completely eliminated protease-resistance of purified PrP^Sc^ species under our experimental conditions (Fig. 6B). As a control, we added the same concentration of an unrelated protein (Bovine Serum Albumin, BSA) and the results did not show any effect on PrP^Sc^ (Fig. 6B). A similar, albeit less pronounced effect, was observed with PrP^Sc^ associated to other murine prion strains, such as 79A and 301 C (Supplementary Figure 2). To determine whether the effect of GRP78 over PrP^Sc^ was time-dependent, 8 μM of the recombinant chaperone was incubated with purified PrP^Sc^ preparations for various times (Fig. 6C). We observed a time-dependent reduction of PrP^Sc^ signal in PK-resistant assays. However, GRP78 activity was optimum only after prolonged incubation. Our results suggest that GRP78 has a direct effect on preventing PrP^Sc^ replication through altering the biochemical/structural properties of PrP^Sc^ into conformations that are sensitive to PK.

## Discussion

Many neurodegenerative diseases are associated with the accumulation of misfolded proteins in the brain^29^,^30^. Compelling evidence has shown that disease-related proteins alter distinct aspects of the secretory pathway, triggering as a common event ER stress. PrP^Sc^ accumulation in prion diseases has been shown to cause an imbalance in ER homeostasis and activation of the UPR in different experimental systems, possibly due to altered calcium homeostasis^3^,^31^. GRP78 is a master regulator of ER protein folding, in addition to fine-tuning the threshold to activate the UPR. Under physiological conditions, GRP78 binds the luminal domains of the three main UPR stress sensors including ATF6, PERK and IRE1 in order to keep them in an inactive state^7^. When misfolded proteins accumulate in the ER, GRP78 preferentially assists folding releasing ER stress sensors, triggering a global UPR response to restore proteostasis^14^.

It has been proposed that chronic ER stress produced by the persistent and progressive accumulation of misfolded proteins leads to massive perturbation of GRP78 and its signaling targets, triggering apoptosis and neurodegeneration^18^. Depletion of GRP78 in Purkinje cells accelerates neuronal degeneration and retards growth in young mice^32^. Similarly, mutant knock-in mice for *GRP78* undergo age-dependent neurodegeneration^33^. In other studies, reduction of *GRP78* expression resulted in increased aggregation and toxicity of proteins harboring poly-glutamine repeats^34^. Dysfunction of GRP78 also deteriorated retinal damage and induced apoptosis in the retinal tubular injury^35^. The disruption of the GRP78 co-chaperone, SIL1, caused ER stress and accumulation of abnormally folded proteins, leading to neurodegeneration linked to apoptosis and autophagy^36^. Similarly, SIL1 has recently been shown to mediate neuroprotection in models of amyotrophic lateral sclerosis^37^. GRP78 associates with caspase-7 and −12 in the ER compartment and inhibits caspase activation and caspase-mediated cell death^38^. Deletion or missense mutation in the ATP binding domain of GRP78 fail to bind caspase-7, leading to loss of its protective effects^39^. GRP78 also exerts an anti-apoptotic effect through suppressing other ER localized apoptotic machinery^40^. These findings provide evidence that the expression level and activity of GRP78 are essential for neuroprotection, to prevent protein aggregation and to regulate proper UPR signaling.

Although ER stress and the UPR appear to be heavily involved in prion disease pathogenesis^3^,^4^, the exact mechanisms are presently unclear. Early studies from our group and others suggested a role for ER chaperones on prion diseases, particularly the disulfide isomerase ERp57/Grp58 which is highly induced in prion infected mice and patients affected with CJD^8^,^21^,^41^. We previously reported that ERp57/Grp58 has a neuroprotective role against PrP^Sc^ toxicity and also physically interacts with PrP^C^ (Ref. 21). ERp57/Grp58 is part of the calnexin and controls the steady state of PrP^C^ levels *in vitro* and *in vivo*^42^. Recent findings suggest that over-activation of PERK signaling lead to sustained eIF2α phosphorylation with concomitant reduction in protein synthesis of a cluster of synaptic proteins^9^. Furthermore, treatment of scrapie infected mice with a PERK inhibitor significantly delayed disease progression^26^. In contrast, we reported that targeting XBP1, the downstream transcription factor of IRE1 does not alter prion pathogenesis *in vivo*^43^. Similarly, caspase-12 deficient animals develop prion disease in the same way as wild type animals^44^. These data suggest that the contribution of the UPR to prion diseases is complex and may depend on the signaling branch affected^11^.

GRP78 plays a dual role, binding hydrophobic fragments of misfolded or unfolded proteins to regulate their folding and prevent their aggregation, and modulating the UPR in acute stress conditions^18^. We hypothesize that GRP78 may have a direct effect in PrP^Sc^ replication and their disease associated phenotypes due to a direct inhibitory activity on PrP misfolding. Our *in vivo* data support this hypothesis, since animals expressing lower levels of GRP78 developed prion disease at a significantly shorter time. From the *in vivo* results, however, it is not possible to distinguish whether GRP78 directly alters prion replication or the effect observed in mice was due to an indirect activation of signaling pathways.

It has been shown that GRP78 can bind disease-associated misfolded proteins and prevent their pathological consequences^16^. For example, over-expressed GRP78 formed a complex with α-synuclein, resulting in reduced toxicity and increased survival of nigral dopaminergic neurons^45^. To gain more detail information about the mechanism by which GRP78 is protective in prion diseases, we performed various studies using prion infected cells as well as cell-free *in vitro* experiments. Reduction of GRP78 in cells led to a significant increase on PrP^Sc^ replication. Conversely, overexpression of *GRP78* reduced PrP^Sc^ formation in cells. Interestingly, incubation of PrP^Sc^ with purified recombinant GRP78 resulted in a significant and dose-dependent decrease of protease-resistant PrP^Sc^. Indeed, after prolonged incubation with an optimal concentration of GRP78, protease-resistant PrP^Sc^ virtually disappeared from the sample. This data suggests that GRP78 may modulate the misfolding and aggregation of PrP^Sc^ by direct interaction with prion proteins. However, given the multiple and important functions of GRP78 we cannot rule out that *in vivo* GRP78 might be acting through various simultaneous mechanisms to attempt preventing prion replication.

Our results may have implications for the development of novel therapeutic approaches against prion diseases. Strategies to enhance the activity of GRP78 have already been tested in other models with successful results^46^,^47^. For example, gene therapy to deliver GRP78 into dopaminergic neurons resulted in reduced α-synuclein aggregation and delayed disease progression in genetic models of Parkinson´s disease^45^. In addition, a small molecule termed BiP protein Inducer X (BIX) increases GRP78 expression *in vivo* and protects neurons in models of brain ischemia^48^,^49^, retinal damage^50^, and photoreceptor death^35^. Furthermore, a series of small molecules and other gene therapy strategies have been developed to target the UPR and reduce ER stress levels on a variety of preclinical models of disease^51^. Altogether, our results indicate that GRP78 may play an important role in the defense against prion propagation. Our findings suggest that modulation of GRP78 activity might provide a novel strategy to design therapies directed to combat prion diseases and maybe other neurodegenerative diseases associated to the accumulation of misfolded protein aggregates.

## Methods

### Infectivity studies

*In vivo* infectivity studies were done in *GRP78*^+/−^ heterozygous (n = 5) or *GRP78*^+/+^ (WT, n = 5) female mice both with C57Bl6 background. *GRP78*^+/−^ mice were previously generated and characterized as described^22^. Animals were 90 days old at the time of prion inoculation. Mice were injected stereotaxically into the right hippocampus with 2 μL of a 10% w/v brain extract obtained from a symptomatic RML-infected mouse. Animals were checked for appearance of prion-associated clinical signs as previously described^52^. Symptomatic animals were sacrificed at the terminal stage of the disease and brains collected for biochemical and histological analyses. Surgical procedures were performed under isoflurane anesthesia. All animal manipulations were carried out in accordance to NIH regulations and approved by the Animal Welfare Committee of the University of Texas Health Science Center at Houston.

### Histopathological studies

Brain tissue was fixed in Carnoy fixative, dehydrated, and embedded in paraffin. Serial sections (7 μm thick) from each block were deparaffinized, hydrated and stained with hematoxylin-eosin (H&E). Then, samples were dehydrated, cleared in xylene and coverslipped with DPX. Samples were visualized with a DMI6000B Leica^®^ microscope. Brain vacuolation profile was determined on H&E stained sections by scoring the extent of vacuolation in midbrain, hypothalamus, thalamus, hippocampus and motor cortex as previously described^24^,^25^.

### PrP^Sc^ purification from infected brains

PrP^Sc^ was purified from the brain of clinically ill mice infected with RML prions, as previously described^53^,^54^. Briefly, brain tissue was homogenized at 10% w/v in PBS. After a low speed centrifugation to remove debris, samples were mixed with 1 volume of 20% sarkosyl and subjected to a series of differential centrifugations employing a Beckman TL-100 ultracentrifuge (OptimaMAX Ultracentrifuge, Beckman-Coulter) with the final step consisting of a sucrose gradient. The resulting material was treated with proteinase K (PK) (100 μg/ml) for 2 h at 37 °C followed by ultracentrifugation to precipitate PrP^Sc^. The purity of PrP^Sc^ was confirmed by silver staining and estimated to be >95%. PrP^Sc^ concentration was measured by micro BCA protein assay reagent (Pierce).

### Proteinase K digestion

Cell lysates or 10% w/v brain homogenates were prepared in PBS supplemented with a cocktail of protease inhibitors as previously described^55^. Debris-cleared aliquots were digested with 50 μg/mL PK with shaking (450 rpm in an Eppendorf thermomixer) at 37 °C for 1 h. PK resistant PrP^Sc^ was then detected by Western blot.

### Western blot

Cell lysates and brain homogenates were prepared in PBS supplemented by a cocktail of protease inhibitors. Protein concentration was determined with the Pierce BCA Protein Assay kit (Thermo Scientific). Proteins from cell lysates or brain homogenates were fractionated by electrophoresis using 4–12% SDS-polyacrylamide gels (SDS-PAGE), transferred into nitrocellulose membranes, and probed with the following antibodies: anti-prion 6D11 antibody (1:5000, Sigma), anti-calreticulin (1:1000, Cell Signaling), anti-GRP94 (1:1000, Cell Signaling), anti-protein disulphide isomerase (anti-PDI, 1:1000, Cell Signaling), anti-protein kinase RNA-like endoplasmic reticulum kinase (PERK) (1:1000, Cell Signaling), anti-phospho-PERK (1:1000, Cell Signaling), anti-inositol-requiring 1 protein (IRE1) (1:1000, Cell Signaling), anti-eIF2α (1:1000, Cell Signaling), anti-phospho-eIF2α (1:1000, Cell Signaling), anti-calnexin (1:1000, Cell Signaling), anti-CCAAT/-enhancer-binding protein homologous protein (CHOP) (1:1000, Cell Signaling), and anti-β-actin (1:1000, Cell Signaling). The immunoreactive bands were analyzed using the Quantify One (4.6.7) software (BioRad^®^).

### Cell culture

Prion infected CAD5 cells (a generous gift from Dr. Charles Weissmann, Scripps Institute, Jupiter, FL) were cultured in Optimem supplemented with 10% fetal calf serum and antibiotics. Cells were maintained in DMEM (N2) or Optimem (CAD5) and split 1:10 at confluence. Mouse embryonic fibroblasts (MEFs) were isolated following a previously described protocol^56^. Briefly, 14 day-old wild type mice embryos were obtained from the uterine horns from pregnant mothers. Head and red organs were removed and the rest was digested in presence of trypsin and DNase, followed by pipette dissociation. Thereafter, trypsin activity was inactivated with MEFs culture medium (DMEM, containing 10% fetal bovine serum, L-glutamine, and penicillin-streptomycin). Cells were centrifuged at low speed (300 × g for 5 minutes) and resuspended in MEFs culture medium. Cells were plated onto flask coated with 0.2% gelatine for 2 h. The fibroblasts are the only cells that have the ability to attach to the gelatine-coated flasks. Cells were grown in MEFs culture medium until they reached 70% confluence.

### Plasmids and siRNA

GFP DNA fragments were removed from pEGFP (Clontech^®^), resulting in pE vector. *GRP78* PCR fragments were subcloned into pE vector to create pE-*GRP78*. Scrambled siRNA as a negative control and siRNA targeting *GRP78* were designed and purchased from Thermo Scientific/Dharmacon. For siRNA transfection, cultures of CAD5 at 70% confluence were transfected with 25 nmol/L scrambled siRNA as a negative control or siRNA targeting *GRP78* using the Lipofectamine^®^ 2000 Reagent (Life technologies).

### Expression and purification of recombinant GRP78

*E. coli* cells [BL21 (DE3)] containing pQE80-*GRP78* were grown overnight in 50 mL of LB medium with carbenicillin (100 μg/ml). The overnight culture was used to inoculate 1 L of LB containing carbenicillin (100 μg/mL) at 37 °C with shaking (250 rpm). After 3 h, 1 mM IPTG (isopropyl-β-d-thiogalactopyranoside) was added to induce protein expression for 4 h. *GRP78* in frame with the N-terminal 6 × His tag sequence was purified under native conditions using Ni-nitrilotriacetic acid (NTA) Superflow resin (Qiagen) using manufacturer’s suggested protocol. After this procedure the protein was highly pure as analyzed by coomassie staining and western blot (Supplementary Figure 3).

### Immunocytochemistry

For double staining of PrP and GRP78, fibroblasts were fixed for 30 minutes in 10% formalin solution. Cells were first pre-treated with sodium citrate and then sequentially incubated with mouse anti-6H4 (1:100) primary antibody and then rabbit anti-GRP78 (1:200) primary antibody overnight, followed by the corresponding Alexa488 anti-mouse secondary antibody (1:500) and Alexa568 anti-rabbit (1:500). Sections were cover-slipped with mounting medium containing DAPI and examined under a confocal laser microscope (Nikon A1R). The degree of overlapping pixels of 6H4 and GRP78 signal was quantified using the NIS-Elements software (Image Analysis System). The intensity of a given pixel in the green image was used as the x-coordinate of the scatter plot and the intensity of the corresponding pixel in the red image as the y-coordinate. Results were displayed in a pixel distribution scatterplot or fluorogram and Pearson’s correlation and Mander’s overlap coefficients were determined.

### Coimmunoprecipitation

Wild type brain homogenates were prepared in PBS at 10% w/v, supplemented by a cocktail of protease inhibitors. Immunoprecipitation was performed with the anti-GRP78 antibody and anti-rabbit IgG Dynabeads or anti-PrP (6D11) and anti-mouse IgG Dynabeds, following the recommendations of the manufacturer (Life Technologies). The presence of PrP or GRP78 in the precipitated material was evaluated by Western blots, as described above. Controls were done using Dynabeds conjugated with the secondary antibody without the primary antibody.

### Statistical Analysis

Means are presented with their standard errors and compared by one-way analysis of variance (ANOVA) followed by Tukey’s multiple comparison test or by two-tailed unpaired t-test with Welch’s correction. For infectivity experiments, Log-rank (Mantel-Cox) test was used to determine differences among the groups. Data was analyzed using the GraphPad prism software, version 5.0. Statistical differences were considered significant for values of P < 0.05.

## Additional Information

**How to cite this article:** Park, K.-W. *et al*. The Endoplasmic Reticulum Chaperone GRP78/BiP Modulates Prion Propagation *in vitro* and *in vivo. Sci. Rep.* **7**, 44723; doi: 10.1038/srep44723 (2017).

**Publisher's note:** Springer Nature remains neutral with regard to jurisdictional claims in published maps and institutional affiliations.

## Supplementary Material

Supplementary Figures
