# Supplementary material for: The Endoplasmic Reticulum Chaperone GRP78/BiP Modulates Prion Propagation in vitro and in vivo
Source: Sci Rep. 2017 Mar 23;7:44723. doi: 10.1038/srep44723 (PMC5363067; doi:10.1038/srep44723)

SUPPLEMENTARY INFORMATION

The Endoplasmic Reticulum Chaperone GRP78/BiP Modulates Prion Propagation *in vitro* and *in vivo*

Kyung-Won Park, Gyoung Eun Kim, Rodrigo Morales, Fabio Moda, Ines Moreno-Gonzalez, Luis Concha-Marambio, Amy S. Lee, Claudio Hetz and Claudio Soto

SUPPLEMENTARY FIGURE 1: Expression levels of PrP^{C} in cells treated with GRP78 siRNA. CAD5 cells transfected with *GRP78* siRNA or control siRNA were harvested and lysed. The level of PrP^{C} before proteinase K treatment was analyzed by Western blotting. The graph depicts the densitometric analysis of 3 replicated blots as the one shown in the left. Values correspond to the average ± standard error. Statistical differences were analyzed by using student's t-test.

SUPPLEMENTARY FIGURE 2: GRP78 reduces the amount of protease-resistant PrP^{Sc} *in vitro* from various murine prion strains. Brain homogenates from terminally ill animals infected with the 301C or 79A prion strains were incubated with different concentrations of recombinant GRP78 for 20hr (1200 minutes). The amount of PrP^{Sc} remaining resistant to PK digestion was assessed by Western blotting. Numbers in the top represent GRP78 concentrations, expressed as µM.

SUPPLEMENTARY FIGURE 3: Purification of recombinant GRP78. A: The degree of GRP78 purity after different steps during the purification procedure was analyzed by electrophoresis on a 4–12% SDS-polyacrylamide gels (SDS-PAGE) stained with Coomassie Blue. Lane 1, total lysate; lane 2, supernatant after centrifugation; lane 3, pellet after centrifugation; lanes 4-10, Elution fractions 1-6 of Ni-nitrilotriacetic acid (NTA) Superflow column. **B**: Western blotting with different amount of purified recombinant GRP78. MWM: molecular weight markers.

Supplementary figure 1

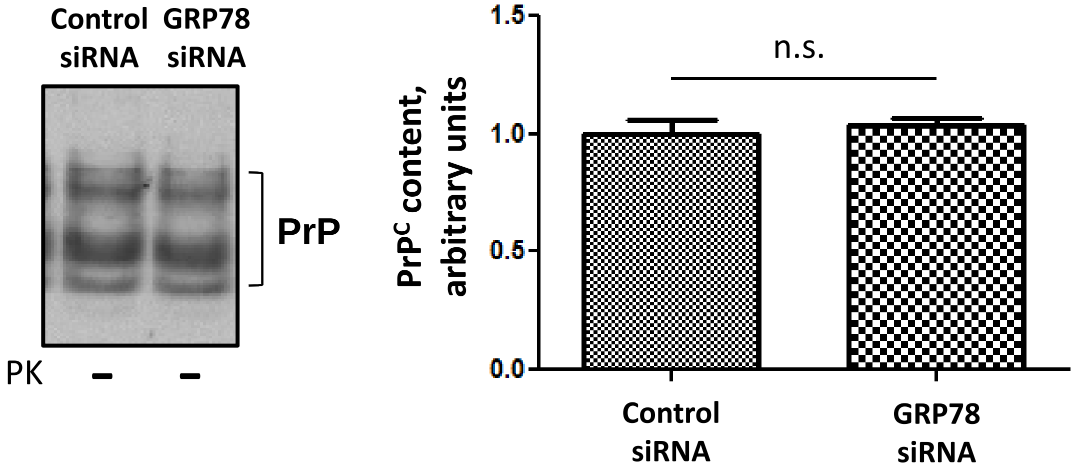

Supplementary figure 2

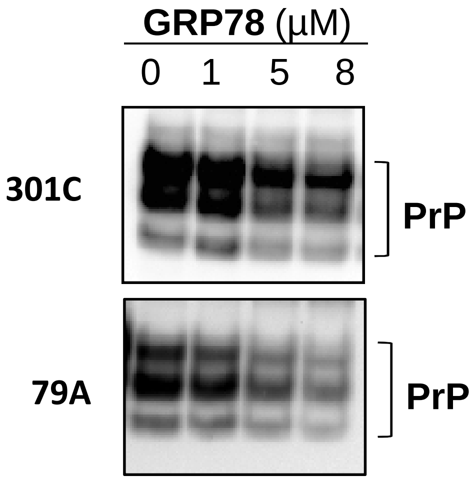

Supplementary figure 3

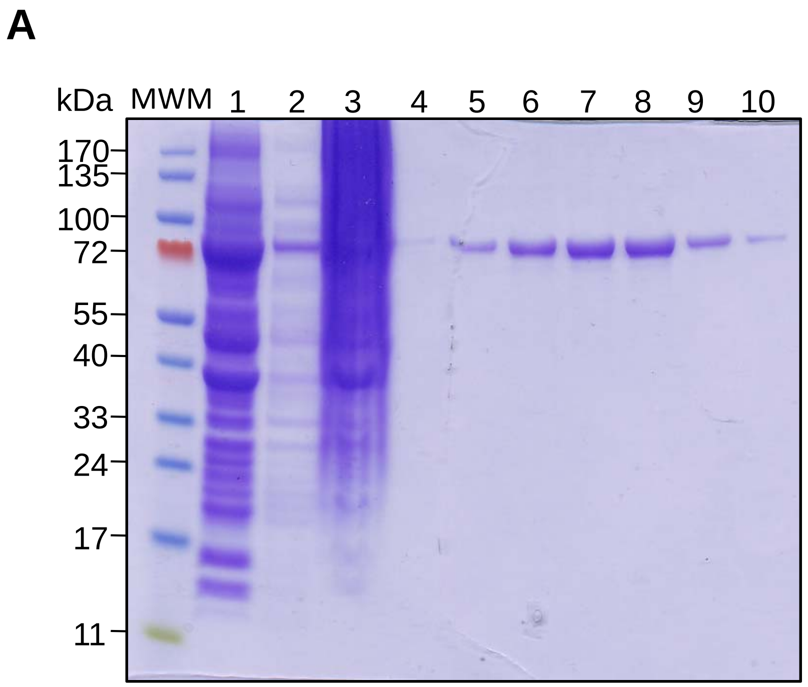

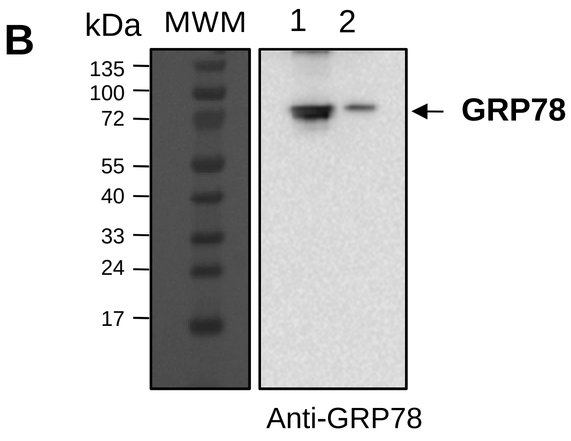

Supplement: Supplementary Figures [file srep44723-s1.pdf]
